# Roles of Oxidative Stress and Autophagy in Alcohol-Mediated Brain Damage

**DOI:** 10.3390/antiox14030302

**Published:** 2025-02-28

**Authors:** Leon Ruiter-Lopez, Mohammed A. S. Khan, Xin Wang, Byoung-Joon Song

**Affiliations:** 1 Section of Molecular Pharmacology and Toxicology, National Institute on Alcohol Abuse and Alcoholism, Bethesda, MD 20892, USA; 2 Department of Neurosurgery, Brigham and Women’s Hospital, Harvard Medical School, Boston, MA 02115, USA; mkhan5@bwh.harvard.edu (M.A.S.K.); xwang2@bwh.harvard.edu (X.W.)

**Keywords:** autophagy, ethanol, alcohol metabolism, brain, oxidative stress, mitophagy, neurotoxicity, neurodegeneration, antioxidants

## Abstract

Excessive alcohol consumption significantly impacts human health, particularly the brain, due to its susceptibility to oxidative stress, which contributes to neurodegenerative conditions. Alcohol metabolism in the brain occurs primarily via catalase, followed by CYP2E1 pathways. Excess alcohol metabolized by CYP2E1 generates reactive oxygen/nitrogen species (ROS/RNS), leading to cell injury via altering many different pathways. Elevated oxidative stress impairs autophagic processes, increasing post-translational modifications and further exacerbating mitochondrial dysfunction and ER stress, leading to cell death. The literature highlights that alcohol-induced oxidative stress disrupts autophagy and mitophagy, contributing to neuronal damage. Key mechanisms include mitochondrial dysfunction, ER stress, epigenetics, and the accumulation of oxidatively modified proteins, which lead to neuroinflammation and impaired cellular quality control. These processes are exacerbated by chronic alcohol exposure, resulting in the suppression of protective pathways like NRF2-mediated antioxidant responses and increased susceptibility to neurodegenerative changes in the brain. Alcohol-mediated neurotoxicity involves complex interactions between alcohol metabolism, oxidative stress, and autophagy regulation, which are influenced by various factors such as drinking patterns, nutritional status, and genetic/environmental factors, highlighting the need for further molecular studies to unravel these mechanisms and develop targeted interventions.

## 1. Introduction

Excessive alcohol (ethanol) consumption causes severe consequences for human health, usually negatively affecting multiple organ systems and contributing to a variety of diseases. According to the National Institute on Alcohol Abuse and Alcoholism (NIAAA), excessive alcohol intake causes more than 200 diseases and accounts for a significant global burden of disease, with millions of individuals affected annually by alcohol-related morbidities and mortalities. In the United States alone, approximately 28.9 million people aged 12 and older reported alcohol use disorder (AUD) in 2023, with annual economic burdens of more than USD 250 billion [1].

Because of its high water solubility, ethanol is distributed to virtually all organs, including the digestive organs, liver, brain, etc., and negatively affects them after heavy drinking. The brain is particularly vulnerable to the toxic effects of alcohol, partly due to having very low levels of antioxidants and antioxidative enzymes and high levels of lipids compared to those in the peripheral tissues such as the liver. Chronic alcohol consumption is associated with various neurodegenerative conditions, including alcohol-related nervous system damage or neurodegeneration, including dementia, Wernicke–Korsakoff syndrome, and fetal alcohol spectrum disorders (FASD) [2,3,4,5]. For example, FASD, a condition resulting from fetal and/or prenatal alcohol exposure, leads to severe developmental and cognitive impairments [6,7,8]. In fact, neuronal cells in gestational periods are much more vulnerable to ethanol-mediated neurotoxicity than adult tissues, eventually leading to FASD [9].

Although the mechanisms underlying alcohol-induced neurodegeneration vary by pattern of alcohol intake such as frequency and amount, nutritional states, gender, genetic makeup, and age, they share several common pathways of toxicity. Key mechanisms include the production of reactive oxygen species (ROS) and reactive nitrogen species (RNS); accumulation of cytotoxic acetaldehyde and other reactive lipid aldehydes, such as 4-hydroxynonenal (4-HNE), malondialdehyde (MDA), and acrolein; induction of endoplasmic reticulum (ER) stress with accumulated misfolded proteins; and impaired lysosomal autophagy or mitophagy upon alcohol exposure [10,11,12]. In addition, alcohol intake alters various signaling pathways including activation of the cell death pathway, elevated inflammation, and mitochondrial dysfunction with depletion of energy supply [13]. Among these, oxidative stress seems to play a central role in initiating inflammation and exacerbating cellular damage, since intake of antioxidants or nutritional support with a balanced diet usually reduces the degree of alcohol-related organ damage in experimental models [14,15,16,17].

Oxidative stress arises from an imbalance between the production of ROS and the cell’s ability to detoxify these reactive molecules [18]. In the context of alcohol metabolism, elevated ROS levels can suppress the function of subcellular organelles, including mitochondria and ER, which results in oxidative modification and inactivation of components in the mitochondrial electron transport chain (ETC), such as complex I (ubiquinone-dependent NADH-oxidoreductase). This modification and inactivation of the ETC produces more electron leakage and ROS, further causing mitochondrial dysfunction and an impaired energy supply, ER stress with elevated levels of misfolded proteins, and eventual cell death. In addition, in the presence of high alcohol concentrations, the ethanol-inducible cytochrome P450-2E1 (CYP2E1) and its isozymes, present in the ER and mitochondria [19,20], also participate in the production of ROS, which causes oxidative modifications to cellular macromolecules, including the proteins, lipids, and DNA. These changes lead to protein post-translational modifications (PTMs), lipid peroxidation products, and DNA-adducts, all of which can potentially contribute to tissue damage, mutagenesis, or carcinogenesis if not treated properly [21]. One notable consequence of oxidative stress is the bi-directional regulations of the lysosomal autophagic pathways [22,23]. Some reports showed that ethanol can activate autophagy, a highly conserved process to remove the damaged proteins or subcellular organelles, while others demonstrated that ethanol can also impair autophagic process through elevated oxidative stress, depending on the patterns of alcohol intake (e.g., chronic versus acute alcohol exposure), cellular contexts, nutritional status, redox balance states, etc. Since autophagy generally serves a protective function by mitigating oxidative damage, its dysregulation can have opposing effects, leading to increased inflammation and cell/tissue injury. In this review, we summarize the current evidence available regarding the involvement of the autophagic pathways in alcohol-related neurotoxicity and their relationship with oxidative stress.

## 2. Materials and Methods

A literature search was conducted on the impact of alcohol exposure on autophagy and oxidative stress in the brain. Investigators LRL and BJS performed the literature search. We searched PubMed until Nov 21, 2024, using the following search terms: “autophagy” and “oxidative stress” or “redox” or “reactive oxygen species” or “cellular stress” or “antioxidants” and “Wernicke-Korsakoff*” or “behavioral*” or “cognitive*” or “neuro degeneration” or “brain*” and “alcohol*” or “ethanol*.” All articles were in English. The search strategy was supplemented with references found through the snowball technique to obtain information from relevant papers available.

Articles discussing autophagy and oxidative stress in response to alcohol consumption in the brain were selected. Papers with mouse models, cell lines, and human samples were considered. However, studies that did not directly evaluate the effects of alcohol on the brain in respect to the mechanisms of autophagy pathways were excluded. Methodological details and molecular effects on autophagy and oxidative stress of each study were extracted, and the information was summarized in different tables.

## 3. Literature Review

### 3.1. Regulation of Autophagy and Lysosomal Protein Degradation

Proteostasis is a normal process to maintain the proper balance of many cellular proteins by regulating the rates of new protein synthesis, adequate protein folding/misfolding, and degradation of cellular proteins [24]. Due to the critical nature of these functions, abnormal proteostasis is frequently associated with many disease states such as alcohol-associated liver disease, immune disorders, cancer, and neurodegeneration [25,26,27]. In mammals, two main protein degradation systems exist to regulate protein homeostasis and maintain their functions and levels. The first one is the ubiquitin-dependent proteasome-dependent degradation system, which is involved in the degradation numerous proteins that are abnormally misfolded in the ER. The other major protein degradation system is autophagic lysosomal proteolysis, which is responsible for the degradation of damaged subcellular organelles and/or aggregated proteins. Thus, disrupted proteostasis often results in accumulation of abnormally misfolded or aggregated proteins, which are potentially toxic to the cells, leading to cell death and various disease states, including alcohol-mediated brain injury and neurodegeneration [27,28].

**Figure 1:**
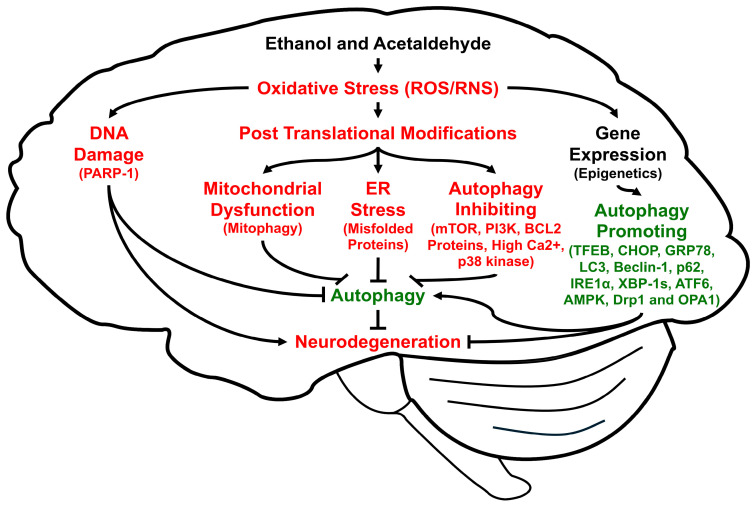
Schematic diagram of the multiple pathways to show how ethanol and acetaldehyde regulate autophagy through elevated oxidative stress, leading to alcohol-mediated neuronal injury and neurodegeneration. Lines with arrowheads indicate stimulation of the pathway while short perpendicular lines represent inhibition.

Autophagy is a highly conserved cellular procedure that removes damaged subcellular organelles and proteins to be re-utilized for promoting cellular homeostasis and survival, especially during insufficient energy supply like fasting [29] or disease states that lead to autophagic flux and autophagic degradation, depending on the nature of disease [30,31,32,33]. Autophagy is usually very low in normal physiological states. However, it can be activated or induced under stressful conditions such as decreased energy supply, hypoxia, ischemia/reperfusion, and viral infections to overcome unfavorable conditions [34], for an excellent review of the autophagy processes, factors, and underlying mechanisms, while it can be suppressed by high levels of oxidative stress and long-term alcohol intake. In fact, autophagy can be regulated by many factors, including oxidative stress, which can cause oxidative DNA damage, alter gene/epigenetic expression, and promote PTMs (Figure 1). Changes in oxidative protein modifications and expression, or single nucleotide polymorphisms of the specific autophagy-related proteins (ATGs) and many associated genes [35], respectively (a few of which are exemplified in Figure 1), can result in different rates of mitophagy, ER stress, and autophagy.

It is also known that specific types of autophagy exist in different cell compartments for the proper disposal of cellular debris, aggregated proteins, and damaged subcellular organelles, such as mitochondria (mitophagy), ER (reticulophagy), peroxisomes (pexophagy) [36], and ribosomes (ribophagy) [37], although we only briefly described the functions of mitophagy and reticulophagy in this review. Thus, abnormal changes in the ubiquitin-dependent proteasomal activities and the lysosomal autophagy are frequently associated with many disease states, including alcohol- or nonalcohol-associated chronic liver diseases, cancer, and aging-related neurodegenerative disorders. Alcohol-associated neuronal injury and behavioral and cognitive impairments [38,39,40] are also known to result from elevated oxidative stress and abnormal regulations of autophagy. For instance, it is known that small amounts of ROS activate autophagy or mitophagy to prevent greater amounts from being produced in the mitochondria and thus providing protection from tissue injury. In contrast, large amounts of ROS (e.g., after chronic excessive alcohol intake) are known to impair autophagy process possibly through phosphorylation, Sirt1-dependent deacetylation, and other PTMs of many proteins involved in the autophagy machinery [34].

Autophagy also repairs DNA damage, which is elevated by oxidative stress or acetaldehyde [41]. In addition, DNA damage causes autophagy where PARP-1 is involved [42,43]. Thus, if autophagy is inhibited, DNA damage cannot be repaired, and the cells undergo apoptosis (instead of re-utilization of the cellular components via autophagy).

### 3.2. Increased Oxidative Stress in the Alcohol Metabolism in the Brain

The majority of consumed alcohol (ethanol) is known to be metabolized by oxidative and non-oxidative pathways in the liver as well as the stomach [44,45]. During the oxidative metabolism pathway, alcohol is oxidized by the cytosolic alcohol dehydrogenase (ADH) to acetaldehyde, which is further oxidized to acetate by the mitochondrial aldehyde dehydrogenase-2 (ALDH2) by using NAD^+^ as a cofactor for both enzymes, resulting in a redox change. In addition, in the presence of a high ethanol concentration through chronic alcohol intake, CYP2E1 present in the endoplasmic reticulum and mitochondria [19,46,47,48] is induced and becomes involved in ethanol oxidation by using NADPH as a cofactor and produce a superoxide anion, which further leads to the production of other reactive oxygen and nitrogen species (ROS/RNS). During the non-oxidative pathway, ethanol is conjugated with various small molecules, such as fatty acids, to produce fatty acid ethyl esters (FAEEs) and phosphatidylethanol [49,50,51].

In general, the oxidative ethanol metabolism increasing the NAD^+^/NADH ratio and inducing CYP2E1 activity [52] is known to cause oxidative stress and tissue injury through activation of the cell death pathways and elevated production of the cytotoxic acetaldehyde and highly reactive lipid aldehydes such as 4-HNE, MDA, and acrolein-adducts [53]. This organ damage occurs in many peripheral tissues and the central nervous system (CNS). Recent studies also showed that non-oxidative ethanol metabolism is involved in tissue injury through elevated ER stress and cell death pathways in the peripheral tissues, including the adipocytes and liver [54].

Unlike the liver, the brain does not contain the classical oxidative ethanol metabolism pathway starting with the ADH, since all five ADH isozymes, including the major ethanol-metabolizing ADH-II, is absent or very low in the brain tissues [55]. In fact, other scientists reported that the majority of alcohol in the brain is known to be metabolized by catalase (~60%) and partially by CYP2E1 (~20%) and others, although the induction of CYP2E1 occurs after chronic alcohol intake [56,57,58,59,60]. However, it is important to consider the potential impacts of the elevated CYP2E1 on the oxidative cell and brain injury, since CYP2E1 was shown to be induced by 17.5 mM ethanol in primary neuronal cells [61], primary astrocytes [62], and rodent brains after chronic alcohol exposures [63,64,65,66].

In addition to these enzymes, ethanol is also known to be metabolized via the non-oxidative pathway to produce ethanol fatty acid esters, which are also known to cause neuroprotection [67,68] or mitochondrial dysfunction and neurotoxicity [49,50,69], depending on the moiety of the fatty acids and suppression of the oxidative ethanol metabolism enzymes [70]. For instance, FAEEs including docosahexaenoic acid (DHA) and eicosapentaenoic acid (EPA) were reported to prevent neurotoxicity or neurodegeneration in animal models of Alzheimer’s disease [71], Parkinson’s disease [67], and Huntington’s disease [72]. On the other hand, ethyl oleate or ethyl palmitate produced through the non-oxidative ethanol metabolism could take place in various tissues, including the pancreas, heart, and brain, where the oxidative ethanol metabolism is weak and causes injury in those tissues [49]. In the latter cases, the amounts of FAEEs positively correlate with the levels of blood alcohol and may promote tissue injury by releasing the free fatty acids, which subsequently suppress the mitochondrial functions, leading to increased organ damage [69].

Finally, most of the ROS produced in the brain could result from the suppressed mitochondrial function and abnormal changes in mitochondrial fission and fusion, which are associated with many chronic disease states, including aging-related neurodegenerative diseases [73,74,75,76]. Excessive amounts of alcohol intake are also known to promote the mitochondrial dysfunction, leading to elevated electron leakage from the mitochondrial ETC to generate ROS and ultimately increased oxidative stress in the peripheral tissues and brain, as reported by many laboratories [77,78,79]. For instance, mitochondrial complexes I–III and IV subunit activities are suppressed by alcohol exposure in rodent models [80]. In addition, alcohol intake is known to alter the intracellular Ca^2+^ balance, cause changes in the mitochondrial Ca^2+^ levels, and alter the dynamics of mitochondrial fusion and fission, leading to mitochondrial dysfunction and neuroinflammation [81,82,83]. All these changes eventually result in elevated oxidative stress and subsequent neurotoxicity.

### 3.3. Effects of Increased Oxidative Stress on Autophagy and Neuronal Damage in Alcohol-Exposed Experimental Models and Individuals with AUD

#### 3.3.1. Effects of Increased Oxidative Stress on Autophagy and Neuronal Damage

In general, the brain usually consumes oxygen at higher rates, possesses high lipid contents, and contains a much smaller amount of antioxidants, including reduced glutathione (GSH), and antioxidative enzymes such as superoxide dismutase (SOD) isozymes and catalase, as well as proteases, compared to those in the liver [84,85,86]. Consequently, various brain cells, including neurons and astroglial cells, are thought to be more sensitive to oxidative injury or cytotoxic agents acetaldehyde and lipid aldehydes, leading to mitochondrial dysfunction and ER stress with misfolded proteins, after exposure to chronic excessive alcohol and other neurotoxic agents compared to the liver hepatocytes. In addition, it is known that an injured liver can regenerate rapidly to fully recover its functions within a short period of time relative to the brain cells [87,88]. The oxidative ethanol metabolism via ethanol-induced CYP2E1, albeit a small amount and with less involvement in the cerebral oxidative ethanol metabolism than catalase, and can cause oxidative stress, neuroinflammation, impairment autophagy, and apoptosis of neuronal cells, leading to brain damage in specific regions, including the hippocampus, cerebellum, and brainstem [61,62,65]. Alcohol-mediated neuronal cell- and/or region-specific damage can result from the unequal distributions of pro-oxidant CYP2E1 [89] and antioxidant ALDH2 [90] in the brain. In addition, hydrogen peroxide (H_2_O_2_) can be generated during the turnover of neurotransmitters such as dopamine and serotonin, contributing to oxidative stress, if not properly managed [91]. Additionally, immune-cell-derived NADPH oxidase can contribute to produce oxidative stress in the brain, leading to ER stress, neuroinflammation, and neurodegeneration in the animal models [92,93] and people with AUD [94]. Furthermore, acetaldehyde generated from oxidative ethanol metabolism, mitochondrial dysfunction, and ER stress can produce additional levels of ROS, Ca^2+^ imbalance, and mitochondrial dynamic changes in the brain, contributing to inhibition of autophagy/mitophagy accompanied by increased neuroinflammation and neurodegeneration in ethanol-exposed cells in rodents [41,95].

Alcohol-mediated oxidative stress is known to cause neurotoxicity and altered autophagy responses possibly through multiple mechanisms as described below. Alcohol-induced changes in autophagic responses seem to be dependent on the different embryonic stages, frequency and patterns of ethanol exposure such as acute and chronic alcohol intake, which causes oxidative stress, various experimental models, nutritional status of the host cells/animals, etc. However, because of the often conflicting results, we have listed many recent reports on the effects of acute and chronic alcohol intake on autophagy regulation in rodent models in Table 1 and Table 2, respectively. In continuation, we have also described the recent reports on the effects of alcohol exposure on autophagy in various brain cell culture models, including neurons, microglia, and astrocytes in Table 3. To provide clinical relevance of these experimental results, we also describe a summary of the effects of ethanol on autophagic flux in the brains of people with AUD (Table 4).

#### 3.3.2. Effects of Increased Oxidative Stress on ER Stress and Neuronal Damage

Under increased oxidative and nitrosative stress, we expect that many oxidative PTMs, such as disulfide oxidation, mixed disulfide formation with glutathione, S-nitrosylation, nitration, phosphorylation, acetylation, and protein-adducts, including acetaldehyde-adducts [121,122,123,124], can take place. These oxidative PTMs can take place in virtually all subcellular organelles of the cytoplasm, ER, mitochondria, and nuclei, contributing to accumulation of oxidatively-modified and/or misfolded proteins, ER stress [125], mitochondrial dysfunction, and epigenetic regulations [126], respectively. Increased oxidative/nitrosative stress and subsequent PTMs also cause blood–brain barrier (BBB) destruction, neuroinflammation, and neurotoxicity [127,128,129,130]. For instance, daily ethanol exposures increased the levels of AGE-albumin [131], histone modifications [132], nitration or acrolein-adducts [133], phosphorylated Tau proteins [134], and amyloid beta accumulation with cognitive impairments [134,135,136].

Reticulophagy is a specific autophagy process where damaged ER with misfolded proteins is engulfed and then degraded by lysosomes. The damaged ER with many misfolded proteins under oxidative stress can result from inactivation of many ER chaperone proteins such as Hsp90, Grp78, and protein disulfide isomerase (PDI). Under normal conditions, these chaperone proteins are responsible for various modifications like glycosylation and disulfide formation for proper folding of their client proteins. However, these chaperone proteins can also be oxidatively modified, and their functions or activities become inhibited under oxidative stress conditions after exposure to alcohol or other neurotoxic agents. For instance, nitration of Hsp90 caused its inactivation, leading to death of motor neurons [137]. Phosphorylation of Grp78 became inactivated in the transformed cells, contributing to suppression of glycosylation with decreased binding with its client protein immunoglobulin heavy chains [138], ER stress with protein misfolding, and cellular damage [139,140]. In the case of alcohol exposure, PDI becomes oxidatively modified [141] and inactivated [142] (Moon KH et al. [122], unpublished results), possibly resulting in decreased binding with or misfolding of the substrate proteins, ultimately contributing to chronic liver disease or neurodegeneration, as extensively reviewed [143].

#### 3.3.3. Effects of Increased Oxidative Stress on Mitophagy and Neuronal Damage

Special autophagy in mitochondria (mitophagy) is known to protect against oxidative stress, mitochondrial dysfunction, inflammation, and aging-related diseases since it not only removes damaged mitochondria but also regulates the rates of neuroinflammation and cognitive deficits [97,112,144,145,146]. Earlier reports showed that Parkin, an E3 ubiquitin ligase, is involved in removing damaged mitochondria [147], while PINK1 (PTEN-induced putative serine/threonine kinase 1) is stabilized on damaged mitochondria to stimulate Parkin, which regulates the cell quality control system by breaking down unneeded or damaged proteins [148]. Thus, PINK1-KO or Parkin-KO mice are thought to have impaired mitophagy compared to the wild-type (WT) mice, and these KO mice become more sensitive to mitochondrial dysfunction and tissue injury caused by alcohol [38,110,149] and other potentially other neurotoxic agents [150]. Additionally, Lin et al. recently showed that chronic alcohol exposure promoted impairment of both receptor-mediated and PINK1-related mitophagy in the medial prefrontal cortex, leading to elevated NLRP3-related neuroinflammation and cognitive decline in C57BL6/J mice through suppression of an antioxidant transcription factor nuclear factor erythroid 2-related factor 2 (NRf2) [40]. In this report, si-RNA mediated silencing of PINK1 or BNIP3 caused mitochondrial dysfunction and alcohol-induced neuroinflammation in BV2 microglial cells. However, treatment with RTA-408, an NRF2 activator, attenuated NLRP3-related neuroinflammation and mitophagy suppression, leading to improvement of alcohol-mediated cognitive dysfunction. These results indicate that alcohol-mediated oxidative stress plays an important role in suppressing mitophagy, which leads to NLRP3 neuroinflammation and cognitive impairment in mice.

In contrast, acetaldehyde induces mitophagic responses with elevated levels of PINK1, Parkin (a member of the E3 ubiquitin ligase, cell quality control system by breaking down unneeded or damaged proteins), resulting in mitochondrial dysfunction and decreased mitochondrial mass, and cytotoxicity in acetaldehyde-exposed SH-SY5Y cells. In this case, the levels of LC3-II, Beclin1, autophagy-related protein Atg5, and Atg16L1 were elevated, while p62 levels were decreased. However, treatment with an autophagy inhibitor such as chloroquine and 3-methyladenine (3-MA), or an antioxidant NAC, prevented decreased mitochondrial mass, suggesting a role of oxidative stress in acetaldehyde-mediated excessive mitophagy [41].

#### 3.3.4. Effects of Increased Oxidative Stress on Neuroinflammation, NETosis, and Neuronal Damage

Excessive alcohol intake also causes neuroinflammation and NETosis in the brain through alterations of the functions of neutrophils. Neutrophils are white blood cells that play a pivotal role in innate and adaptive immunity, wound healing, the resolution of inflammation, and fight against infection from various pathogens [151,152]. The polymorphonuclear neutrophils (PMNs) are involved in several types of non-inflammatory events, e.g., senescence, apoptosis, and efferocytosis [153,154,155], as well as inflammatory processes, e.g., necroptosis, ferroptosis, and necrosis [156,157,158], including NETosis [159], while they defend the host against the inflammatory threat or infection. In many organs, especially in the brain, these types of cell death can contribute to secondary brain damage by causing a high-grade neuroinflammation.

Like other types of cell death, NETosis is also modulated by autophagy/mitophagy [154,155]. It also mediates a few molecular pathways through the oxidative stress, which activates signaling molecules such as NF-κB, NADPH oxidase, Protein Kinase C (PKC), cytokines (IL-1β, IL-8, and HMGB1), anti-neutrophilic cytoplasmic autoantibody (ANCA), all-trans-retinoic acid (ATRA), transient receptor potential channel M2 (TRPM2), etc. [160,161].

More importantly, autophagy regulates the formation of NETs/NETosis. During NETosis, autophagy can manipulate neutrophil cell death to resolve the inflammation in several organs by regulating neutrophil degranulation, differentiation, metabolism, and the formation of neutrophil extracellular traps (NETs) against the pathogens and dreadful stimuli. The active steps and sequence of events in the molecular pathways that occur during the coordination between the autophagy and induction of NETs/NETosis are very crucial in deciding the fate of the cells in which the mTOR/REDD1 (regulation in development and DNA damage response 1) pathway is the central regulator of autophagy in these granulocytes [162]. It has been shown that internal stimuli within the neutrophils activate the protein-arginine deiminase 4 (PAD4) enzyme, which is a common mediator in multiple signaling pathways, controlling the execution of the formation of NETs and occurrence of NETosis. In contrast, inhibition of the PAD4 enzyme significantly blocks the process of NETosis [163]. Alcohol can induce and inhibit autophagy/mitophagy, which modulates the formation of NETs and regulation of NETosis in various organs including the brain, depending on the severity of acute or chronic conditions, amounts of alcohol consumption, and host conditions. The autophagy process in neutrophils regulates not only neutrophil metabolism during granulopoiesis but also the formation of NETs and NETosis, which are closely associated with neuroinflammation and neuronal injury.

This section updates the advancement of the relationship between autophagy and not only intrinsic changes in neutrophil per se but also extrinsic regulation of the events, involving NETs formation/NETosis in response to acute and chronic alcohol consumption. For example, alcohol-exposed neutrophils produce several inflammatory danger molecules in their intracellular space as well as around their microenvironment in the brain. These pro-inflammatory environment prime neutrophils to form NETs/NETosis, which leads to autophagy-mediated generation of ROS and superoxide from NADPH oxidase activity, degranulation, and an increase in calcium levels to activate the PAD4 enzyme, influencing myeloperoxidase/elastase and histone citrullination activities for epigenetic regulation. Thus, understanding the molecular and cellular mechanisms involving the processes of autophagy and the formation of NETs/NETosis will identify potential targets to develop anti-alcohol drugs for clinical application and provide treatment options to patients with alcohol misuse.

#### 3.3.5. Effects of Increased Oxidative Stress on Autophagy and Neuronal Damage by Regulating the Cell Signaling Pathways

As mentioned before, chronic or binge alcohol intake causes oxidative stress, leading to neuroinflammation and organ damage mainly through suppressing antioxidants and defensive enzymes with activation of pro-oxidant proteins and mitochondrial dysfunction possibly by oxidative PTMs. Alcohol-mediated oxidative stress can alter the activities of many protein kinases and phosphatases that are involved in the cell signaling pathways by the regulation of many proteins in ER stress, neuroinflammation, neurogenesis, and neurotoxicity [164,165,166,167,168,169]. For instance, the activities of mTOR kinase, AMPK/mTOR, PI3K/Akt/mTOR, mitogen activated protein kinases, PKC isoforms, and GSK-3β, known to be directly and indirectly involved in the regulation of autophagy-related proteins and genes, including ATG12, LC3-II, Beclin-1, p62, and Bcl-2 (Figure 1), are modulated by alcohol-mediated oxidative stress [101,170,171,172,173,174,175,176]. Furthermore, ethanol exposure can activate the TLR4-NF-κB-cytokines pathway, which can lead to impaired autophagy accompanied by elevated neuroinflammation and neuronal injury [101,111,118,135]. Although the underlying mechanisms by which ethanol-mediated oxidative stress can alter the rates of autophagy and neuronal injury are incompletely understood, the changes in autophagy responses seem to be dependent on the patterns of ethanol exposure such as the frequency, amount, and acute or chronic alcohol intake as well as nutritional status of the host cells/tissues.

### 3.4. Potential Therapeutic Agents Against Autophagy in Alcohol-Exposed Brains

Alcohol is eliminated from the brain much more slowly than from other organs, and alcohol accumulates in the largest amount in brain tissue compared to other organs [102]. Current medications for AUD are insufficiently effective, highlighting the need for novel highly effective therapies [177,178]. Autophagy plays an important role in neurodegenerative diseases [179], and modulating autophagy by melatonin or its precursor as a therapeutic strategy offers a promising potential against alcohol-related brain damage.

Since autophagy exhibits both a protective mechanism and a damage pathway related to programmed cell death [180], we and others have reported that autophagy can be a double-edged sword in the context of stroke-induced brain injury [180] and aging-related neurodegenerative diseases [179], as well as alcohol-mediated brain damage [181]. On one side, autophagy provides a protective mechanism against alcohol-induced brain damage, and ethanol treatment increased mTOR activity and decreased expression of several ATG genes including Atg12, Atg5, p62/SQSTM1, and LC3 [10]. In this case, autophagy enhancers/inducers likely enhance autophagic flux in alcohol-exposed brains by clearing damaged organelles or misfolded proteins. In contrast, we and others have reported that autophagy acts as a double-edged sword, contributing to brain damage, as seen in the context of stroke-induced brain injury [180,182] and alcohol-related brain damage [181]. Ethanol exposure enhances autophagy markers like Map1lc3-II (LC3-II) and Beclin-1 expression while decreasing SQSTM1 (p62) expression in the brain, liver, and neuroblastoma cells [97,183].

This dual nature of autophagy presents opportunities for targeted therapies. Enhancing autophagy with enhancers/inducers can protect against alcohol-induced brain damage, while inhibitors may mitigate harmful autophagy activation. In addition, targeting molecular pathways such as mTOR, AMPK, and PI3K/Akt/mTOR offers promising therapeutic strategies [102,170,171,172,173,174,175,176]. This review discusses potential therapeutic agents for modulating autophagy in alcohol-exposed brains, as exemplified in Table 5, Table 6 and Table 7.

In addition, many other naturally occurring polyphenol flavonoids and NRF2 activator sulforaphane can prevent oxidative stress, leading to activation of autophagy and improvement of alcohol-associated liver injury, as extensively reviewed [198]. Since many small-molecule plant-derived polyphenols or flavonoids, like quercetin, luteolin, rutin, berberine, and curcumin, are known to have antioxidant effects and pass through the BBB [199,200,201,202,203,204,205,206,207,208], they can also be used for protecting against alcohol-mediated oxidative stress, impaired autophagy, and neurotoxicity.

## 4. Conclusions

In this review, we have briefly described various types of alcohol-mediated brain injury and neurodegeneration, literature search methods, alcohol metabolism in the brain, sources of oxidative stress, general properties and types of autophagy, and potential translational approaches against alcohol-mediated brain damage by regulating the rates of autophagy. However, as we emphasized, the rates of autophagy or mitophagy are differentially affected, depending on the pattern (binge or chronic) of alcohol intake, nutritional status, and other environmental and genetic factors, all of which affect various cell signaling pathways. Similar factors and underlying mechanisms for impaired autophagy and mitophagy with accumulation of damaged, aggregated proteins, and neuronal damage can be induced by various neurotoxic agents and aging-related neurodegenerative disease states [202,203,204,205,206,207]. A key distinction between alcohol-mediated brain damage and other neurodegenerative diseases could be the selective activation of CYP2E1 by alcohol, since CYP2E1 is not induced by aging-related neurodegeneration. Regardless of distinguished pathophysiological conditions, one common factor could be increased oxidative stress (Figure 1), which will negatively affect the downstream autophagic processes, leading to impaired autophagy and neurodegeneration. Due to the complexity of autophagy regulations, further molecular studies are warranted. For instance, we expect to see additional studies on oxidative PTMs of the individual proteins involved in the different steps of autophagy and their functional alterations. Based on the molecular mechanistic studies on alcohol-mediated autophagy regulations, additional therapeutic agents against alcohol-induced neurotoxicity as well as other brain diseases can be identified and developed for clinical usage in the future.

## Figures and Tables

**Table 1:** Autophagy in acute alcohol exposure in animal models.

First Author, Year	Models and Methods	Summary of Effects
Alimov et al., 2013. [96]	C57BL/6 mice were injected subcutaneously with saline or ethanol (2.5 g/kg, 20% solution in saline) twice at 0 h and 2 h	Injection of ethanol induced neuroapoptosis on postnatal day 4 (PD4). However, little effect was observed in the brain of PD12 mice. Expression of genes that regulate UPR and autophagy was significantly higher on PD12 than PD4.
Chen et al., 2012. [97]	C57BL/6 mice were injected subcutaneously with saline or ethanol (2.5 g/kg, 20% solution in saline) twice at 0 and 2 h	Ethanol increased oxidative stress and neuronal death. By activating autophagy through the mTOR pathway with agents like rapamycin, some of the ethanol-mediated effects were attenuated.
Boschen et al., 2023. [98]	Pregnant C57BL/6J mice were exposed to a single dose of 2.9 g/kg ethanol, as an FASD model	Ethanol exposure during neurulation disrupts gene expression in the rostroventral neural tube. This affects pathways involved in metabolism, cell cycle, and organogenesis. Such deficits can contribute to developmental malformations in the brain and craniofacial structures.
Uguz et al., 2024. [99]	Wistar albino female rats (weighing between 250 and 350 g and 8–12 weeks old) were exposed to ethanol (18% *v*/*v*) via oral gavage. The total volume prepared was 2 mL, and the total dose administered was 1 g/kg body weight. Ethanol gavage was administered twice per week during the second and third weeks of pregnancy.	Acute alcohol exposure during pregnancy disrupted gene expression in the neural tube. This also affected developmental pathways, further contributing to craniofacial and brain malformations.
Montesinos et al., 2018. [100]	Thirty-day-old female C57BL/6 wild-type (WT) and TLR4 knockout (TLR4-KO) mice were exposed to morning doses of either saline or 25% (*v*/*v*) ethanol (3 g/kg) on two consecutive days with 2-day gaps without injections for 2 weeks Rapamycin to inhibit the mTOR pathway	Exposure to alcohol impaired autophagy mechanisms by increasing the activity of autophagy inhibitor mTOR, lowering LC3-II levels and accumulating p62. Inhibition of mTOR by rapamycin restored levels of excitatory scaffolding synaptic proteins (PSD-95 or SHANK3), p62, and partly re-established the LC3-II levels. Deletion of TLR4 ameliorated autophagy dysfunctions and decreased the frequency and size of the synaptic connections in ethanol-exposed mice.
Pascual et al., 2021. [101]	C57BL/6 adolescent female and male mice (PND30) were treated with ethanol (3 g/kg) on two consecutive days at 48 h intervals over 2 weeks	Ethanol treatment decreased the density and morphology of dendritic spines. These effects are associated with learning and memory impairments, and changes in the levels of both phosphorylation and miRNAs of the transcription factor CREB were observed. Rapamycin administration, inhibiting mTOR/autophagy dysfunctions, prior to ethanol administration restored ethanol-induced changes in both plasticity and behavior dysfunctions in adolescent mice.
Luo, 2014. [9] (REVIEW)	FASD Model, PD4 vs PD12	Acute ethanol exposure induced a protective autophagic response that helped alleviate oxidative stress and neuronal apoptosis in the developing brain.
Kurhaluk et al., 2020. [102] (REVIEW)		Acute alcohol exposure causes the depletion of the critical antioxidant GSH and a reduction in the GSH/GSSG redox state. The oxidative stress induced by acute ethanol intoxication led to structural and functional impairment in tissues that are indicated by the main marker of lysosomal activity. The plethora of melatonin effects can prevent lysosomal destruction in tissues during ethanol-induced intoxication by limiting the increased activity of lysosomal enzymes and resulting in oxidative stress.
Yang et al., 2015. [103] (REVIEW)		Acute alcohol exposure caused long-lasting effects on emotional and memory deficits, suggesting a functional and structural change in the hippocampus in mice. In PD7 mice, ethanol induced ER stress in the developing brain. In PD4 mice, ethanol induced wide-spread neuroapoptosis. In PD12 mice, ethanol induced little effect on the brain. Expression of pro-apoptotic genes, such as cleaved caspase-3, was much higher in PD4 than PD12. Expressed levels of genes that regulate UPR and autophagy, such as atg6, atg4, atg9, atg10, beclin1, bnip3, cebpb, ctsb, ctsd, ctss, grp78, ire1α, lamp, lc3 perk, pik3c3, and sqstm1, were significantly higher in PD12 than PD4.
Fujii et al., 2021. [104] (REVIEW)	FASD model, PD7 2.5 g/kg 20% saline twice s.c. injection Rat hippocampal slices treated with ethanol or acetaldehyde in the absence or presence of integrated stress response inhibitor	Ethanol exposures caused increased levels of autophagy markers, specifically LC3-II, and oxidative stress indicated by the upregulation of ER stress proteins (GRP78, ATF6, and CHOP) and phosphorylation of eIF2α, PERK, and IRE1α within 4 h in PD7. Ethanol and acetaldehyde impaired memory formation in hippocampal neurons by inducing ER stress. Ethanol and acetaldehyde induced NMDAR activation and synthesis of 5α-reduced neurosteroids, leading to inhibition of long-term potentiation (LTP). This result was prevented by inhibiting integrated stress response (ISR) with ISRIB (ISR inhibitor) and stimulating the liver X receptor with agonist GW3965. These results suggest that ISR and ER stress responses exacerbate ethanol-mediated CNS damage, in contrast to the protective role of ISRIB.
De Ternay et al., 2019. [105] (REVIEW)	Acute ethanol exposure over 8 h for 4 days with CBD as a protective agent	Ethanol exposure led to oxidative stress, increasing neuronal degeneration in the hippocampus and entorhinal cortex. With co-administration of CBD, there was a significant reduction in cell death. CBD’s antioxidant effects were comparable to tocopherol.

**Table 2:** Autophagy in chronic alcohol exposure in animal models.

First Author, Year	Models and Methods	Summary of Effects
Sumitomo et al., 2017. [106]	Ulk1-null mice were exposed to four cycles of chronic intermittent ethanol (CIE, 20% ethanol for 4 days followed by water for 7 days), followed by intraperitoneal (i.p.) injection of ethanol (2 g/kg/body weight, once daily, in 15% solution) for 4 days	Chronic intermittent ethanol exposure downregulated Ulk1-mediated autophagy in the prefrontal cortex, leading to p62 accumulation, impaired exploratory behavior, deficits in object recognition, and reduced voluntary ethanol consumption in these mice.
Davis-Anderson et al., 2018. [107]	Timed pregnant Sprague Dawley rats, as an FASD model	Chronic alcohol exposure during pregnancy altered the fetal brain proteome, which significantly impacted proteins involved in cellular growth, autophagy, oxidative stress, and mitochondrial dysfunction in the hippocampus, cortex, and cerebellum. These changes likely contribute to neurodevelopmental deficits associated with FASD.
Nasef et al., 2021. [108]	Seventy female Swiss albino mice aged 4–6 weeks, weighing 10–15 g, 15% alcohol solution for 55 days with or without simvastatin (10 mg/kg/day)	Chronic alcohol exposure induced neurodegeneration by promoting oxidative stress, inflammation, and protein aggregation. However, simvastatin treatment, especially when started early, mitigated the alcohol effects by improving the redox state, suppressing apoptosis, and promoting autophagy and neurogenesis.
Lu et al., 2020. [109]	Thirty-eight-week-old male Wistar rats were fed either an ethanol-containing liquid diet or an isocaloric pair-feeding control	Chronic ethanol exposure led to liver damage, increased inflammatory cytokines, and impaired autophagy. However, mice fed a diet containing fish oil had improved autophagic activity and were protected against ethanol-induced liver injury by inhibiting the Akt signaling pathway.
Hwang et al., 2017. [110]	Male (4~5 months old, 26–27 g) wild-type (WT) C57BL/6 mice and Park2 KO mice. Within 10 days, ethanol comprised 35.8% of the total calories consumed.	Ethanol exposure in Park2 KO mice exacerbated dopaminergic neurodegeneration by increasing reactive oxygen species, mitochondrial dysfunction, and pro-apoptotic protein expression while inhibiting autophagy and mitochondrial function through p38 kinase activation.
Uguz et al., 2024. [99]	Wistar albino female rats (weighing between 250 and 350 g and 8–12 weeks old) were exposed to ethanol (20% *v*/*v*) via oral gavage, at a total volume of 2 mL and a dose of 4.5 g/kg body weight for 4 weeks	Chronic alcohol exposure leads to increased oxidative stress, altered autophagy signaling in the hippocampus and cortex, and impaired learning ability in offspring. Sex differences were observed, with female mice outperforming males in learning tasks. The expressed levels of IBA1, LC3B, GAD65, and mGluR5 were higher in female rats with chronic alcohol exposure.
Pla et al., 2014. [111]	Male 7-week-old C57BL/6 WT and TLR4 knockout (KO, TLR4^−/−^) mice weighing 18–20 g were maintained with water (WT and KO control) or water containing 10% (*v*/*v*) ethanol, and solid diet ad libitum for 4 months	Ethanol exposure downregulated p62 and other autophagic proteins while further impairing autophagy through inducing the formation of autophagic vacuoles with greater volume density. After alcohol exposure, inhibitor of mTOR, rapamycin administration partially reduced neuroinflammation. TLR4 is upstream in the mTOR activation cascade. Alcohol exposure caused little or no changes in mTOR phosphorylation and the autophagy pathway in TLR4-KO mice.
Bian et al., 2022. [39]	Female Kunming mice exposed to ethanol (4 g/kg/d) or saline for 28 days in the absence or presence of Puerarin (25, 50, or 100 mg/kg, ip)	Ethanol exposure caused cognitive impairment with elevation of p-mTOR/mTOR and suppressed autophagy marker proteins. The middle and high doses of Puerarin prevented these changes and improved cognitive function.
Chen et al., 2024. [112]	Young (3-month-old) and aged (23-month-old) male mice exposed to Gao’s chronic+binge alcohol paradigm or chronic ethanol liquid diet for 4 weeks	Chronic alcohol exposure decreased in the levels of hippocampal transcription factor EB (TFEB), which regulates the expression of lysosomal autophagy-related genes, and spatial memory while increasing the levels of apoptotic cells and aggregated phosphorylated-Tau proteins in young mice but not in aged mice. Thus, natural aging has a greater impact on the rates of autophagic influx and spatial memory decline in mice than chronic alcohol exposure.
Luo et al., 2014. [9] (REVIEW)		Chronic ethanol exposure in mice activated mTOR signaling, leading to impaired autophagy and increased oxidative stress, which exacerbated neuronal vulnerability and neurodegeneration.
Kurhaluk et al., 2020. [102] (REVIEW)	Melatonin treatment	Ethanol exposure likely caused melatonin suppression, leading to desynchronosis (circadian disruption). The circadian timing system can also be related to an altered drinking behavior or ethanol response. Alcohol can alter the circadian rhythm and pace making functions of the suprachiasmatic nuclei. Chronic alcohol consumption also led to a depletion of the critical antioxidant GSH and reduction in the GSH/GSSG redox state. Alcohol exposure also elevated plasma endotoxin levels and activated the hepatic endotoxin signaling cascade. These alcohol-mediated changes could be mitigated by melatonin treatment.
Fujii et al., 2021. [104] (REVIEW)	Mice exposed to water containing 10% (*v*/*v*) ethanol for 5 months	Chronic ethanol exposure increased TLR4 signaling, which results in activation of caspase-1, NLRP3 inflammasomes, and production of IL-1β and IL-18 to induce pyroptosis (cell death).

**Table 3:** Autophagy in alcohol-exposed cell culture models—neurons, microglia, or astrocytes.

First Author, Year	Models and Methods	Summary of Effects
Wang et al., 2023. [113]	Mouse microglia BV-2 cells were treated with different doses of alcohol (0.5 mg/mL, 4 mg/mL, and 10 mg/mL) for 3 h or 12 h, respectively	Alcohol exposure in microglia BV-2 cells disrupted autophagy and promoted apoptosis.
Wu et al., 2012. [114]	HepG2 E47 cells which express CYP2E1 and HepG2 C34 cells which do not contain CYP2E1 were treated with 100 mM ethanol for 8 days. Some cells were also treated with 3-methyladenine (MA, 2.5 mM) or rapamycin (0.2 μg/mL) or Chlormethiazole (CMZ, a CYP2E1 inhibitor, 100 μM) or N-acetylcysteine (NAC, an ROS scavenger, 5 mM).	Ethanol treatment increased fat accumulation and oxidant stress but decreased autophagy in E47 HepG2 cells. These results suggest that ethanol-mediated oxidative stress inhibits autophagy.
Flores-Bellver et al., 2014. [115]	Human retinal pigment epithelial cell line ARPE-19; cells were treated for 24 h at different ethanol concentrations: 80, 200, 400, and 600 mM	Chronic ethanol exposure increased autophagy flux and mitochondrial fragmentation in ARPE-19 cells. Autophagy served as a protective factor in the cells by degrading damaged mitochondria and lowering lipid peroxidation products, such as 4-HNE, although the ethanol concentrations were unphysiologically high.
Bonet-Ponce et al., 2015. [116]	Human retinal pigment epithelial ARPE-19 cells were treated for 24 h with ethanol	Ethanol exposure induced mitochondrial fission and activated autophagy through Drp1 and OPA1 in ARPE-19 cells. Autophagy served a cytoprotective role by removing damaged mitochondria, while mitochondrial ROS drove the autophagic response.
Yan et al., 2022. [41]	Human neuroblastoma SH-SY5Y cells, acetaldehyde exposure	Acetaldehyde-induced cytotoxicity in SH-SY5Y cells triggered oxidative stress and excessive mitophagy. Increased levels of ATGs and mitochondrial degradation were observed after exposure.
Chen et al., 2012. [97]	Human neuroblastoma SH-SY5Y cells obtained from ATCC, 0.4% ethanol	Ethanol exposure caused decreased cell viability and increased oxidative stress, with the involvement of the mTOR pathway in mediating these effects.
You et al., 2024. [117]	Pheochromocytoma line 12 (PC12) cells	Ethanol exposure in PC12 neuronal cells induced mitochondrial fragmentation and dysfunction, activating autophagy during degeneration. PGC-1α-mediated mitochondrial biogenesis was crucial for neurite regrowth and cell survival, which allowed for recovery from this ethanol-induced damage.
Pla et al., 2016. [118]	Cultured astroglial cells, ethanol (50 mM) 0–24 h Cultured neuronal cells, ethanol (50 mM) 0–24 h	In astrocytes, ethanol induces overexpression of several autophagy markers (ATG12, LC3-II, CTSB, and lysosomal cathepsin B) induced via TLR4 pathways. An increased amount of lysosomes in the WT astrocytes created a basification of lysosomal pH and lowered phosphorylation levels of autophagy inhibitor mTOR, along with activation of complexes beclin-1 and ULK1.
Wang et al., 2023. [113]	Microglia BV-2 cells	Modest alcohol consumption activated autophagy. Chronic exposure induced organelle damage, oxidative stress, and affected autophagy function, leading to apoptosis.
Luo et al., 2014. [9] (REVIEW)	Cultured fetal cortical neurons	Ethanol exposure modulated autophagy through pathways involving mTOR and AMP-activated kinase (AMPK), resulting in oxidative stress and endoplasmic reticulum stress triggering neurotoxic effects.
Yang et al., 2015. [103] (REVIEW)	SH-SY5Y neuroblastoma cells and primary cerebellar granule neurons	Exacerbated ER stress (GRP78, CHOP, ATF4, ATF6, and phosphorylated PERK and EIF2a) was observed when ethanol was combined with tunicamycin or thapsigargin. Antioxidants such as GSH and NAC improved ethanol’s stimulation of ER stress and cell death.
Fujii et al., 2021. [104] (REVIEW)	Microglia and monocytic cell line Neuronal cell line Neuroblastoma cell line with a phosphatase inhibitor (Salubrinal)	Mitochondrial ROS specifically induce NLRP3 but not NLRC4. Ethanol exposure caused Golgi fragmentation and disruption of protein transport between ER and Golgi. Ethanol was seen to cause compaction of the Golgi apparatus and interrupt normal neurite growth in developing neurons. Co-administration of Salubrinal with ethanol further exacerbated accumulation of amyloid beta.
Guo et al., 2023. [119] (REVIEW)	Microglia, human peripheral blood mononuclear cells, and the murine macrophage cell line J774	Ethanol may regulate the levels of specific miRNAs, subsequently controlling microglia activation. Ethanol exposure also increased oxidative stress, observed through elevated levels of mitochondrial ROS and inflammatory cytokines like IL-1β, with mitochondrial damage and ROS accumulation contributing to NLRP3 inflammasome activation, particularly in macrophages and microglia.
Aki et al., 2013. [120] (REVIEW)	Neuronal SH-SY5Y cells Various cell lines, ethanol exposure with rapamycin or wortmannin	Ethanol induced mitophagy by suppressing the mTOR pathway and increasing ROS generation, contributing to neuronal damage and stress. Autophagy reduced apoptosis caused by ethanol. The autophagy inducer rapamycin alleviates while the autophagy inhibitor wortmannin aggravates ethanol-induced apoptosis.

**Table 4:** Autophagy in humans with alcohol use disorder.

First Author, Year	Models and Methods	Summary of Effects
Kurhaluk et al., 2020. [102] (REVIEW)	Melatonin treatment	Chronic alcohol intoxication depleted the tissue resources of the pineal gland and leads to marked disturbances in its function. Chronic ethanol exposure also resulted in functional and structural changes in the nervous system that have been associated with learning and memory impairment. Alcohol administration significantly increased lipid and protein oxidation and decreased the activities of antioxidant enzymes.
De Ternay et al., 2019. [105] (REVIEW)	Cannabidiol (CBD)	CBD was protective against alcohol-related liver steatosis and brain damage (cognitive impairment) in individuals with AUD by reducing oxidative stress and stimulating autophagy.
Aki et al., 2013. [120] (REVIEW)		Chronic alcohol intake caused an increase in autophagy in the brain.

**Table 5:** Autophagy enhancers/inducers/mitophagy activators.

Compound	Classification	Summary of Effects
Rapamycin	An mTOR Complex 1 (mTORC1) inhibitor	Rapamycin restores autophagic flux, preventing ethanol-induced cell death and vascular plasticity [32]. Rapamycin inhibits ethanol neonatal effect and normalizes NMDA receptor changes in the hippocampus, the prefrontal cortex, and the striatum of the brain of adult rats [184]. Rapamycin mitigates FASD-related behavioral deficits, improving spatial learning and reducing vulnerability to alcohol addiction [184]. Rapamycin also enhances LC3 lipidation and protects neurons from apoptosis. Inhibition of mTOR by rapamycin restores the levels of p62 and partly re-establishes the LC3-II levels in the prefrontal cortices of ethanol-treated mice [100]. Rapamycin restores mitophagy [97].
Spermidine	Upregulates the expression of autophagy promoting genes, (e.g., ATG5) Modulates the NMDA receptor	Spermidine can reverse the suppression of autophagy-promoting genes caused by oxidative damage and mitochondrial dysfunction. Spermidine facilitates the reinstatement of AUD-induced conditioned place preference/conditioned place preference, involving the polyamine binding site at the NMDA receptor [185].
Metformin	Autophagy enhancer	Metformin provides antioxidant, anti-inflammatory, and neuroprotective effects. Metformin promotes autophagy by increasing autophagosome formation, as evidenced by elevated LC3-II levels. Metformin increases autophagy in the brain by activating AMP-activated protein kinase (AMPK), which subsequently inhibits the mTOR signaling pathway, thereby triggering the process of autophagy [186]. Metformin decreases the expression of p62 in the brain, which is a marker of impaired autophagy, suggesting that metformin promotes autophagy by reducing p62 accumulation in brain tissues [187]. Metformin boosts mitophagy, protecting against alcohol-induced tissue injury [188].

**Table 6:** Autophagy inhibitors.

Compound	Classification	Summary of Effects
Bafilomycin A1 (BafA1)	Inhibitor of autophagosome and lysosome fusion It is a macrolide antibiotic that inhibits the later stages of autophagy	Bafilomycin A1 inhibits lysosomal acidification [189]. Ethanol exposure increases the p62 levels, while BafA1 potentiates ethanol-increased LC3 lipidation [97]. As the inhibitor of autophagy, ethanol exposure impeded the upregulation of LC3 II induced by BafA1 [40].
Piracetam	Nootropic drug Derived from neurotransmitter γ-aminobutyric acid	Piracetam prevents ethanol-induced memory loss by increasing hippocampus long-term potentiation (LPT) and inhibiting hippocampus neuronal apoptosis. Piracetam reduces ethanol-induced neuronal damage by regulation of autophagic action. In more detail, piracetam decreases ethanol-induced LC3-II and Beclin-1 expression, increases the phosphorylation of mTOR, and inhibits Akt phosphorylation [190].

**Table 7:** Selective modulators.

Compound	Classification	Summary of Effects
Erythropoietin (EPO)	Glycoprotein hormone	Intranasally administered EPO promotes remyelination and synapse formation in chronic alcohol-affected neocortex and hippocampus [191]. Exogenous recombinant human rhEPO, which enters the cerebrum of the brain through the intranasal route, activates the EPO receptor and the downstream ERKs and PI3K/AKT signaling and significantly suppresses autophagy-related degradation of NRf2 [191]. These results, thus, highlight autophagy-related Nrf2 activity as the key mechanism mediating the neuroprotective effects of EPO [191].
Wortmannin	Autophagy inhibitor	Wortmannin blocks the formation of autophagosomes and inhibits the PI3K/Akt pathway [192]. Wortmannin attenuates ethanol-promoted LC3 lipidation and LC3 puncta [97]. Wortmannin reverses increased phosphorylation of the PI3K-Akt-GSK3β-CREB pathway during alcohol withdrawal [193].
RTA-408 (omaveloxolone)	NrF2 activator	RTA-408 ameliorates chronic alcohol exposure-induced cognitive impairment by modulating mitophagy in the medial prefrontal cortex of C57BL/6J mice in vivo [40]. RTA-408 improves cognitive impairment in neonatal mice via reducing the apoptosis of hippocampal neurons and activating Nrf2 [194].
Cannabidiol (CBD)	Natural component of cannabis	CBD stimulates autophagy in vitro and in vivo, leading to alleviation of lipid accumulation [105]. CBD stimulates autophagy signaling transduction though crosstalk between the ERK1/2 and AKT kinases [195]. CBD’s neuroprotective, immunomodulatory, and antioxidant properties could prevent or alleviate some alcohol-related brain damage [105].
Hydrogen disulfide (H_2_S)	A gasotransmitter [196]	H_2_S protects against ethanol-mediated oxidative stress, enhanced ER stress, neuronal damage, and neurotoxicity [125]. H_2_S improves spatial memory impairment via increases in BDNF expression and hippocampal neurogenesis in early postnatal alcohol-exposed rat pups [197].

## Abbreviations

The following abbreviations are used in this manuscript:

AUD	Alcohol use disorder
FASD	Fetal alcohol spectrum disorders
ROS	Reactive oxygen species
4-HNE	4-hydroxynonenal
MDA	Malondialdehyde
ER	Endoplasmic reticulum
ETC	Electron transport chain
CYP2E1	Cytochrome P450-2E1
PTMs	Post-translational protein modifications
ATGs	Autophagy-related proteins
ADH	Alcohol dehydrogenase
ALDH2	Aldehyde dehydrogenase-2
FAEEs	Fatty acid ethyl esters
CNS	Central nervous system
DHA	Docosahexaenoic acid
EPA	Eicosapentaenoic acid
GSH	Glutathione, reduced
SOD	Superoxide dismutase
H_2_O_2_	Hydrogen peroxide
PD#	Postnatal day #
WT	Wild-type
KO	Knockout
PC12	Pheochromocytoma line 12 cells
AMPK	AMP-activated kinase
PKC	Protein Kinase C
CBD	Cannabidiol
BBB	Blood–brain barrier
PDI	Protein disulfide isomerase
PINK1	PTEN-induced putative serine/threonine kinase 1
NRf2	Nuclear factor erythroid 2-related factor 2
3-MA	3-methyladenine
PMNs	Polymorphonuclear neutrophils
ANCA	Anti-neutrophilic cytoplasmic autoantibody
ATRA	All-trans-retinoic acid
TRPM2	Transient receptor potential channel M2
REDD1	Regulated in development and DNA damage response 1
NETs	Neutrophil extracellular traps
PAD4	Protein-arginine deiminase 4
BafA1	Bafilomycin A1
EPO	Erythropoietin
H_2_S	Hydrogen disulfide
